# Complete Genome Sequence of Cyanobacterial Siphovirus KBS2A 

**DOI:** 10.1128/genomeA.00472-13

**Published:** 2013-08-22

**Authors:** Alise J. Ponsero, Feng Chen, Jay T. Lennon, Steven W. Wilhelm

**Affiliations:** Department of Microbiology, University of Tennessee, Knoxville, Tennessee, USAa; Institute of Marine and Environmental Technology, University of Maryland Center for Environmental Science, Baltimore, Maryland, USAb; Department of Biology, Indiana University, Bloomington, Indiana, USAc

## Abstract

We present the genome of a cyanosiphovirus (KBS2A) that infects a marine *Synechococcus* sp. (strain WH7803). Unique to this genome, relative to other sequenced cyanosiphoviruses, is the absence of elements associated with integration into the host chromosome, suggesting this virus may not be able to establish a lysogenic relationship.

## GENOME ANNOUNCEMENT

As obligate parasites, viruses can regulate their host population dynamics but also influence the structure and productivity of microbial communities (1, 2). *Synechococcus* species are an abundant and ecologically important group of *Cyanobacteria* found in freshwater and marine ecosystems worldwide. Virus-cyanobacterium interactions may have important implications for global biogeochemical cycles. The most commonly isolated cyanophages are myoviruses and podoviruses (3, 4). Siphoviruses are a third group of viruses that infect cyanobacteria, but they have received less attention (5).

The genomes of 5 cyanosiphoviruses have recently become available: that of P-SS2, a siphovirus infecting *Prochlorococcus* (MIT9313) (6), followed by the cyanosiphoviruses S-CBS1, S-CBS2, S-CBS3, and S-CBS4, isolated from the Chesapeake Bay Estuary, all infecting *Synechococcus* populations (5). Here, we present the complete genome of cyanosiphophage (KBS2A, originally named KBS-S-2A), a virus that infects *Synechococcus* sp. strain WH7803.

The virus was isolated by plaque assay from the Chesapeake Bay by plating on *Synechococcus* sp. WH7803. Purified virus DNA was submitted to the Broad Institute as part of the Marine Phage Sequencing Project, where it was sequenced to ~30-fold coverage using 454 pyrosequencing. Translated open reading frames (ORFs) were compared with known protein sequences using the BLASTp program. ORF annotation was aided by the use of PSI-BLAST, HHpred, gene size, and domain conservation.

The genome size of KBS2A is 40,658 bp. In total, 64 ORFs have been predicted in this genome; of these, 43 have homologues in databases, and among them, 33 have been assigned to a putative function. For most (88%) predicted ORFs with homologues, homology has been found with the other cyanosiphovirus genomes. We compared the genomic arrangements of the 6 sequenced cyanosiphoviruses using dot plot and global gene homology and found no common genomic organization, suggesting strong mosaicism in the cyanosiphoviruses.

In cyanophages, cyanobacterium-related proteins can be found and are often associated with photosynthesis and transcriptional regulation (6). In previously sequenced cyanosiphovirus genomes (5, 6), numerous viral genes (6 to 40 per genome) possess homology with host genes. In the case of the KBS2A genome, only 3 ORFs (coding for RNA polymerase sigma factor RpoD, HNH endonuclease, and a putative DNA polymerase) show such homology, implying less exchange (and potentially interaction) with the host genome.

The first annotated cyanosiphovirus genome (that of P-SS2) showed the presence of genes identified as encoding an integrase and excisionase, which are enzymes that allow for phage integration into the host’s genome (6). Moreover, the annotation of cyanosiphoviruses S-CBS1 and S-CBS3 led to the discovery of a prophage-like structure in two sequenced *Synechococcus elongatus* strains (5). In phage genomes, tRNA genes serve as indicators of potential phage integration by site-specific recombination (7, 8), although recent models have offered alternative suggestions for the role of these genes (9). Sequences of this nature can, however, be found in the P-SS2 and S-CBS4 genomes. No such features (tRNAs, integrases, etc.) were found in the genome of KBS2A, suggesting that this siphovirus might be an exclusively lytic phage rather than a temperate phage.

### Nucleotide sequence accession number. 

The complete sequence of the *Synechococcus* phage KBS2A genome can be accessed under the GenBank accession no. HQ634187.
